# A lightweight MHDI-DETR model for detecting grape leaf diseases

**DOI:** 10.3389/fpls.2024.1499911

**Published:** 2024-12-06

**Authors:** Zilong Fu, Lifeng Yin, Can Cui, Yi Wang

**Affiliations:** College of Rail Intelligent Engineering, Dalian Jiaotong University, Dalian, China

**Keywords:** RT-DETR, target detection, grapevine leaf disease, lightweighting model, deep learning

## Abstract

Accurate diagnosis of grape leaf diseases is critical in agricultural production, yet existing detection techniques face challenges in achieving model lightweighting while ensuring high accuracy. In this study, a real-time, end-to-end, lightweight grape leaf disease detection model, MHDI-DETR, based on an improved RT-DETR architecture, is presented to address these challenges. The original residual backbone network was improved using the MobileNetv4 network, significantly reducing the model’s computational requirements and complexity. Additionally, a lightSFPN feature fusion structure is presented, combining the Hierarchical Scale Feature Pyramid Network with the Dilated Reparam Block structure design from the UniRepLKNet network. This structure is designed to overcome the challenges of capturing complex high-level and subtle low-level features, and it uses Efficient Local Attention to focus more efficiently on regions of interest, thereby enhancing the model’s ability to detect complex targets while improving accuracy and inference speed. Finally, the integration of GIou and Focaler-IoU into Focaler-GIoU enhances detection accuracy and convergence speed for small targets by focusing more effectively on both simple and difficult samples. The findings from the experiments suggest that The MHDI-DETR model results in a 56% decrease in parameters and a 49% reduction in floating-point operations, respectively, compared with the RT-DETR model, in terms of accuracy, the model achieved precision rates of 96.9%, 92.6%, and 72.5% for accuracy, mAP50, and mAP50:95, respectively. Compared with the RT-DETR model, these represent improvements of 1.9%, 1.2%, and 1.2%. Overall, the MHDI-DETR model surpasses the RT-DETR and other mainstream detection models in both detection accuracy and degree of lightness, achieving dual optimization in efficiency and accuracy, and providing an efficient technical solution for automated agricultural disease management.

## 1 Introduction

Grapes are extensively grown across the globe and serve not only as a key ingredient in wine production but also in the production of foods such as sultanas and jams, while being rich in medicinal value Taskesenlioglu et al. (2022). However, grape growth is often threatened by various diseases, with leaf diseases being among the most prevalent and severe issues. Black measles disease, a serious condition that can lead to the weakening or death of grapevines Raynaldo et al. (2024). It affects not only the foliage but also the fruit and vines, leading to substantial economic losses. Black rot, a highly destructive disease in grape production, spreads rapidly in humid conditions, perforating leaves and causing fruit rot, which severely impacts grape yield and quality Szabó et al. (2023). Leaf blight causes extensive dieback of grape leaves, severely disrupting photosynthesis, which reduces grape yield and the nutritional value of the fruit. Prolonged leaf blight decreases plant resistance, making the grapevines more susceptible to other diseases Liu et al. (2020). These diseases not only hinder the growth, development, and yield of grapes but also directly impact their quality and economic returns, resulting in significant losses and challenges for the grape-growing industry Shantkumari and Uma (2023). Traditionally, leaf disease detection has been dependent on manual visual inspection, whereby farmers or professionals visually assess leaves for signs of disease, such as spotting or discoloration Arsenovic et al. (2019). However, this approach tends to be time-consuming, labor-intensive, and costly, while being strongly affected by subjective judgment, professional expertise, and environmental conditions, leading to inconsistent and unreliable diagnostic outcomes Khakimov et al. (2022). In large vineyards, the efficiency and accuracy of manual inspection frequently fail to meet practical demands, leading to the oversight or delayed control of diseases, which, in turn, hinders the stable development of the grape industry.

To address the limitations of traditional detection methods, recent progress in areas such as computer vision, image processing, and machine learning has drawn increasing attention to deep learning-based detection approaches Kotwal et al. (2023). Convolutional Neural Networks (CNNs) are a class of deep learning models specifically designed to process grid-like data, such as images. Spatial features, including edges and textures, are extracted via convolutional layers. Li et al. (2021b) identified the shortcomings of traditional image processing methods, highlighting the significant advantages of deep learning, particularly convolutional neural networks, for improving detection accuracy and efficiency. Unlike traditional detection, which requires labor-intensive manual observation of each leaf, intelligent detection methods powered by deep learning can rapidly scan large numbers of leaves, enhancing efficiency. However, despite these advantages, challenges remain in model generalization and dataset diversity. Shoaib et al. (2023) acknowledged the potential of deep learning techniques in enhancing accuracy and speed in plant disease detection, emphasizing the importance of early detection. Intelligent detection methods can identify subtle symptoms that might otherwise go unnoticed by traditional approaches, which often rely on the naked eye and may miss early signs of disease. Zhu et al. (2021) introduced a technique that integrates super-resolution image enhancement with deep learning, utilizing bilinear interpolation. This method was evaluated on both the Plant Village dataset and images collected from orchards in the field, with experimental results showing improvements of 5.94% in detection accuracy and 10.67% in recall. By applying five data augmentation techniques to expand the image dataset, Pandian et al. (2022) demonstrated that the Conv-5 DCNN achieved an average classification accuracy of 98.41% on the test dataset. These studies indicate that CNNs are especially suitable for large-scale image recognition tasks due to their ability to automatically learn hierarchical feature representations from raw input data Younesi et al. (2024). Their flexibility and scalability render them an optimal solution for plant disease detection in diverse environmental conditions. However, CNNs struggle to effectively capture global contextual information, and techniques such as region suggestion networks and non-maximum suppression, commonly used in target detection, may increase computational burden Gong et al. (2021).

To overcome these difficulties, researchers have begun exploring new model architectures, the most revolutionary of which is the Transformer model. Dosovitskiy et al. (2020) introduced the Vision Transformer (ViT), which utilizes the Transformer framework for image processing. An image is segmented into fixed-size patches, which are then linearly embedded into a sequence that serves as input to a standard Transformer encoder. Although this method accounts for global image relationships and has shown promising outcomes in plant disease recognition, it generally demands a substantial amount of training data to attain higher accuracy. Li et al. (2023). Consequently, combining the strengths of CNNs and Transformers in plant disease detection to extract richer image features has become a key research direction. The DEtection TRansformer (DETR) model, developed by the Facebook team, effectively processes target detection tasks by integrating CNN’s local feature extraction with the Transformer’s global modeling capability Carion et al. (2020). The integration of Transformers and CNNs has been increasingly explored by scholars.For example, Lu et al. (2022) employs the Ghost convolution module as the backbone network for ViT, which first generates a small portion of the primary feature maps and subsequently generates additional feature maps through linear transformations. This approach reduces the model’s parameter count and floating-point operations (FLOPs), thereby lowering memory usage and computational cost. The Ghost-convolution module effectively reduced the computational requirements of the model while maintaining an accuracy of up to 98.14% in the detection of grape leaf diseases and insect pests. Yu et al. (2023) designed the Inception convolution module, which significantly enriches information and enhances ViT’s feature extraction capabilities, conducting experiments on four different plant disease datasets with highly accurate results. ViT has garnered growing attention in the computer vision community, achieving promising results Han et al. (2022); Islam (2022).

As CNNs and ViTs continue to advance in plant disease recognition, accuracy is commonly enhanced by increasing the number of model parameters Wu et al. (2021). However, increasing the number of parameters introduces certain drawbacks, including the necessity for more computational resources during training and inference, as well as limitations in real-time applications. Consequently, plant disease detection is frequently conducted on mobile devices, including embedded systems and smartphones, which encounter challenges such as limited processing capacity, storage, and excessive power consumption. For example, Gole et al. (2023) proposed the lightweight visual transformer network TrIncNet, which achieves high accuracy while simultaneously reducing the number of parameters by replacing the MLP module in ViT with an Inception block and incorporating jump connections. This design makes it particularly suitable for deployment on devices with limited computational resources, significantly reducing both the model’s computational complexity and energy consumption, thus enabling real-time processing on mobile platforms. Similarly, Li et al. (2021a) developed a lightweight RegNet-based method for apple leaf disease recognition, achieving 99.23% accuracy on a small, unbalanced dataset through transfer learning. By reducing the model’s parameter count and focusing on more efficient feature extraction methods, this approach demonstrates high compatibility with low-power devices by reducing memory and processing load without sacrificing accuracy. Li and Li (2022) further introduced the ConvViT structure, merging CNN and Transformer architectures, thereby reducing parameters and FLOPs to 32.7% and 21.7% of those in Swin-Tiny, respectively. This makes ConvViT a practical solution for devices with limited computational capacity while maintaining competitive accuracy.

In general, the above models reduce the number of parameters while maintaining a certain level of accuracy. However, models deployed on mobile or embedded devices must be comprehensively evaluated, particularly in agricultural settings, where there is an increasing demand for low power consumption and low latency under outdoor conditions. Additionally, the algorithms need to exhibit stability to withstand external interferences, such as weather, climate, and varying light conditions. These challenges call for a new deep learning approach that balances efficiency, performance, and external factors to better tackle grape leaf disease issues.This study presents a lightweight method for grape leaf disease detection using an enhanced RT-DETR framework Zhao et al. (2024) to tackle these challenges. The key contributions of this paper are as follows:

1. The generic inverted bottleneck block structure from the MobileNetv4 network is used to replace the residual blocks in the original backbone, reducing computational load and parameter sizes while preserving inference speed, making it more suitable for resource-limited devices.
2. The dilated convolution from the UniRepLKNet network is fused with the RepC3 module to form the DRBC3 module, which replaces the original convolution block. This modification reduces computational resource demands while expanding the sensory field, significantly accelerating inference speed.
3. A novel light-SFPN architecture is introduced by integrating the Hierarchical Scale Feature Pyramid Network with Efficient Local Attention and the DRBC3 module. This architecture enhances the detection capability of small targets while maintaining low computational and parametric requirements, improving inference speed and model convergence.
4. Focaler-IoU is used to dynamically control the model’s focus on difficult and simple samples, combined with GIoU to form Focaler-GIoU. This loss function replaces the original model’s GIoU, resulting in lower loss and faster regression outcomes.
5. A comprehensive evaluation of the grape leaf disease dataset demonstrated that the proposed MHDIDETR architecture surpasses the original RT-DETR framework in both model efficiency and detection accuracy, while delivering significantly improved results over other detection models.

## 2 Materials and methods

### 2.1 Data processing

A total of 4,030 grape leaf samples were selected from the AI Challenger 2018 crop disease dataset. Among them, the four categories of samples, including black measles disease, black rot, leaf blight, and healthy, were categorized as 1,368, 1,129, 1,059, and 473 samples, respectively. Considering the uneven distribution of the samples, the healthy category was augmented to 995 samples using data augmentation techniques, resulting in a total of 4,551 samples. In addition, all images were processed using Python scripts to standardize the image size at 640×640 through bilinear cubic interpolation, As shown in **Supplementary Table S1** of the **Supplementary Materials**.

The 4551 images are randomly partitioned into a training set, a testing set, and a validation set in an 8:1:1 ratio. The sample size for each category in each dataset is provided in **Supplementary Table S2** of the **Supplementary Materials**.

### 2.2 Access to data and description of data

**Figure 1:**
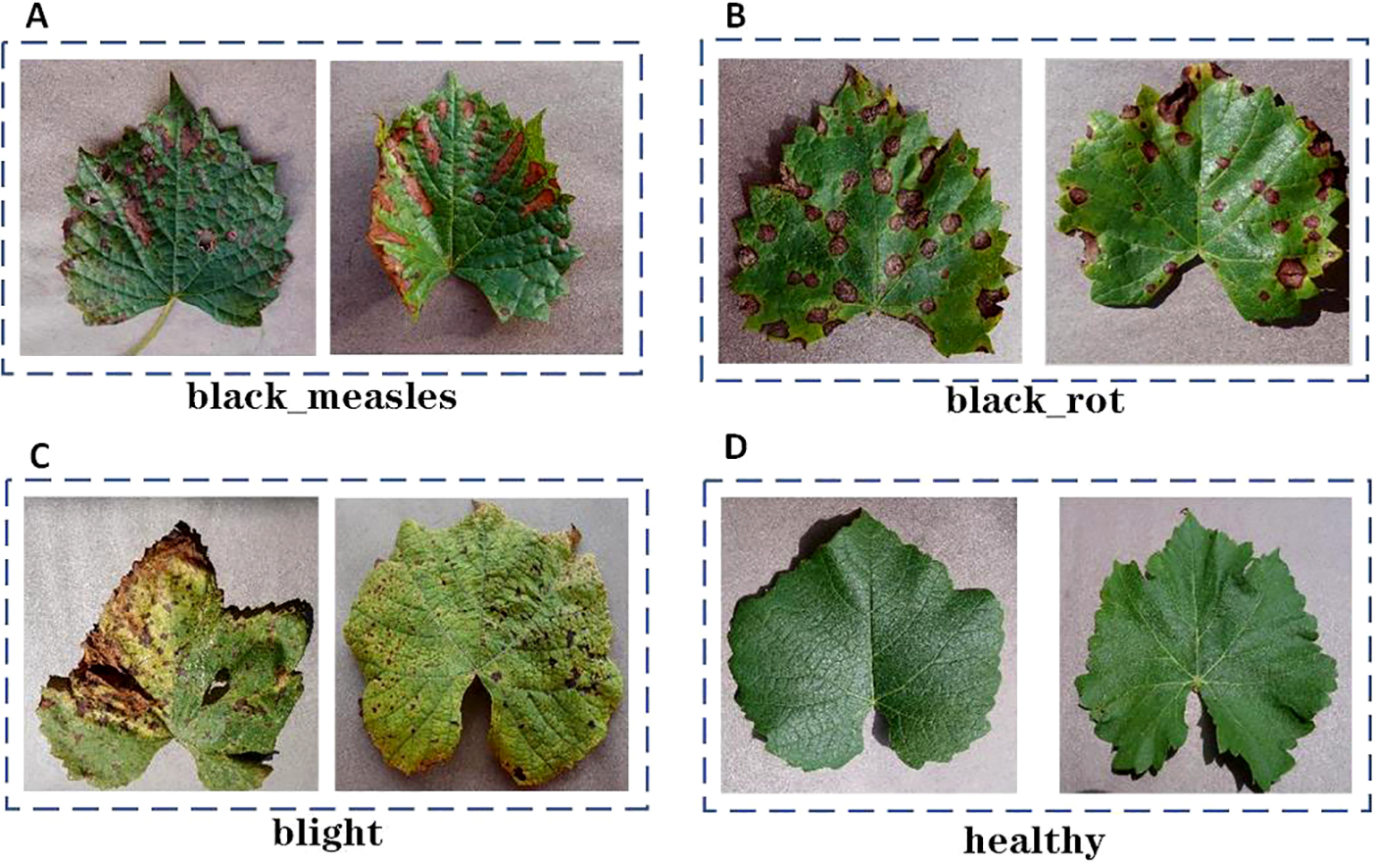
Grape leaf disease example figure. **(A)** black measles. **(B)** black rot. **(C)** blight. **(D)** healthy.

The AI Challenger 2018 crop disease dataset was manually annotated with the LabelImg tool to guarantee precise identification of each sample and enhance the model’s ability to learn broader features, thereby improving its generalization to previously unseen data, as illustrated in **Figure 1**. Following processing, four types of samples were identified: black measles, black rot, leaf blight, and healthy leaves. To improve the model’s robustness, the category of healthy grape leaves was introduced, which presents several advantages. First, the model can learn the features of healthy grape leaves, allowing for more accurate differentiation between healthy and diseased samples. Additionally, comparing healthy and diseased samples enables the model to develop a deeper understanding of infection characteristics, such as changes in color, shape, and texture.

### 2.3 Model selection

RT-DETR Zhao et al. (2024), a novel real-time end-to-end target detector, offers high accuracy and fast inference when identifying a wide range of small diseases in grape leaves. Additionally, its Transformer Jaderberg et al. (2015) architecture enables the model to directly predict final bounding boxes and category scores without the need for additional post-processing to filter or merge overlapping detection boxes. This architecture also preserves the model’s stability.

**Figure 2:**
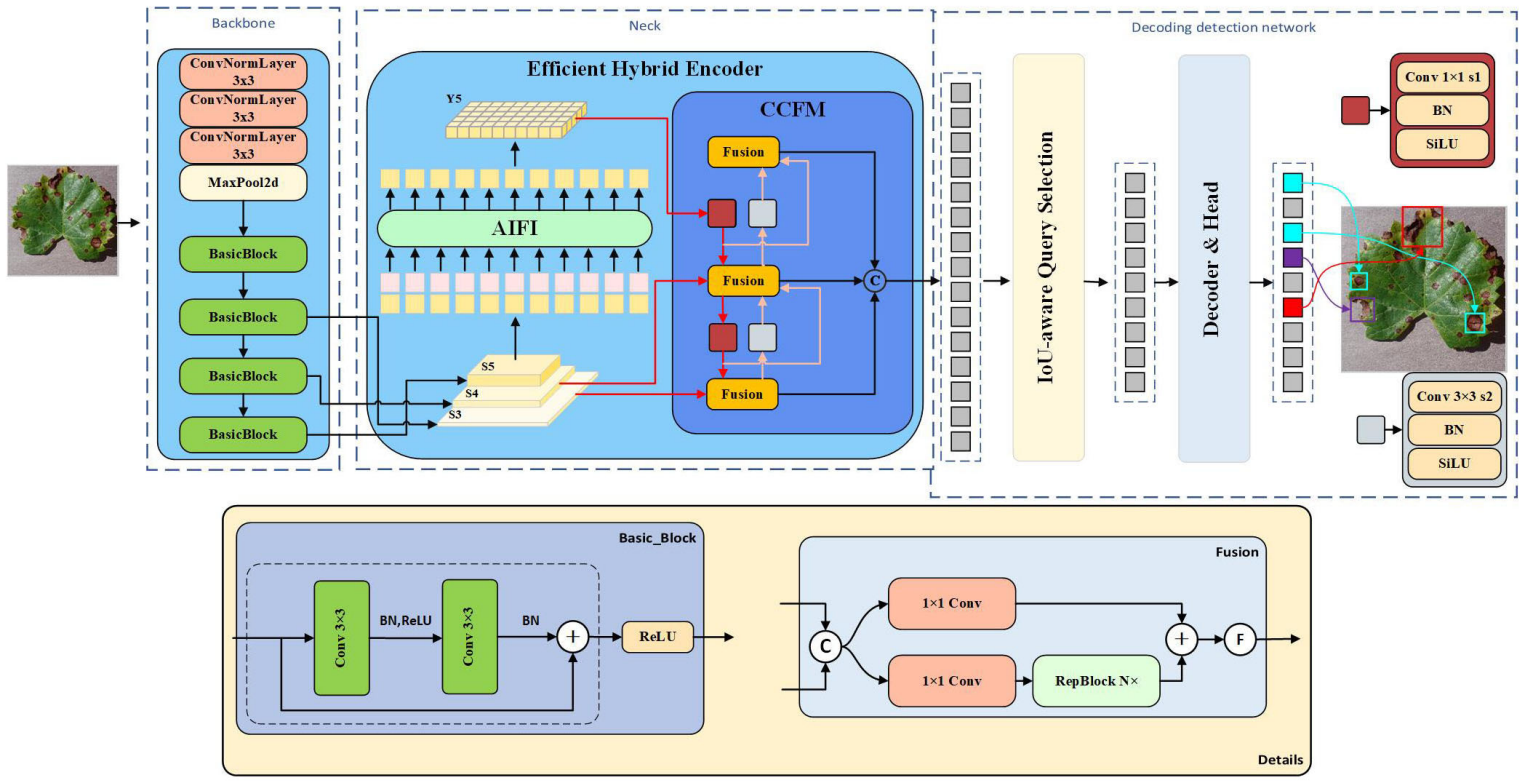
RT-DETR network structure diagram.

In the RT-DETR target detection model, numerous backbone networks are provided, including ResNet He et al. (2016), HGNet, and several others. The ResNet-18 backbone network features a leaner structure and lower complexity compared to other networks. It is well-suited for deployment on resource-constrained devices and grape leaf disease detection, with only a slight reduction in accuracy compared to other models. This achieves a favorable balance between accuracy and computational overhead. Therefore, in this study, the ResNet-18 backbone network is selected as the base model. The architecture first receives an input image, which is processed by a convolutional neural network composed of multiple residual blocks to extract key features. Three different scales of high-level features (S3, S4, and S5) are then extracted into the neck network through varying step sizes and simultaneously serve as inputs to the hybrid encoder. Subsequently, the advanced feature S5 is encoded by intra-scale feature interaction (AIFI), while the processed S5, S3, and S4 enter the cross-scale feature fusion module (CCFM) for multi-scale feature interaction, where they are converted into sequential image features. Finally, these features are passed into the decoder detection network, where IoU-aware querying is employed to select a fixed number of image features from the encoder output sequence as the initial object query. Accurate prediction frames and confidence scores are then obtained through iterative querying. Overall, the combination of CNN and Transformer in RT-DETR presents significant potential for improvement in object detection. The model architecture is depicted in **Figure 2**.

### 2.4 MHDI-DETR model structure

**Figure 3:**
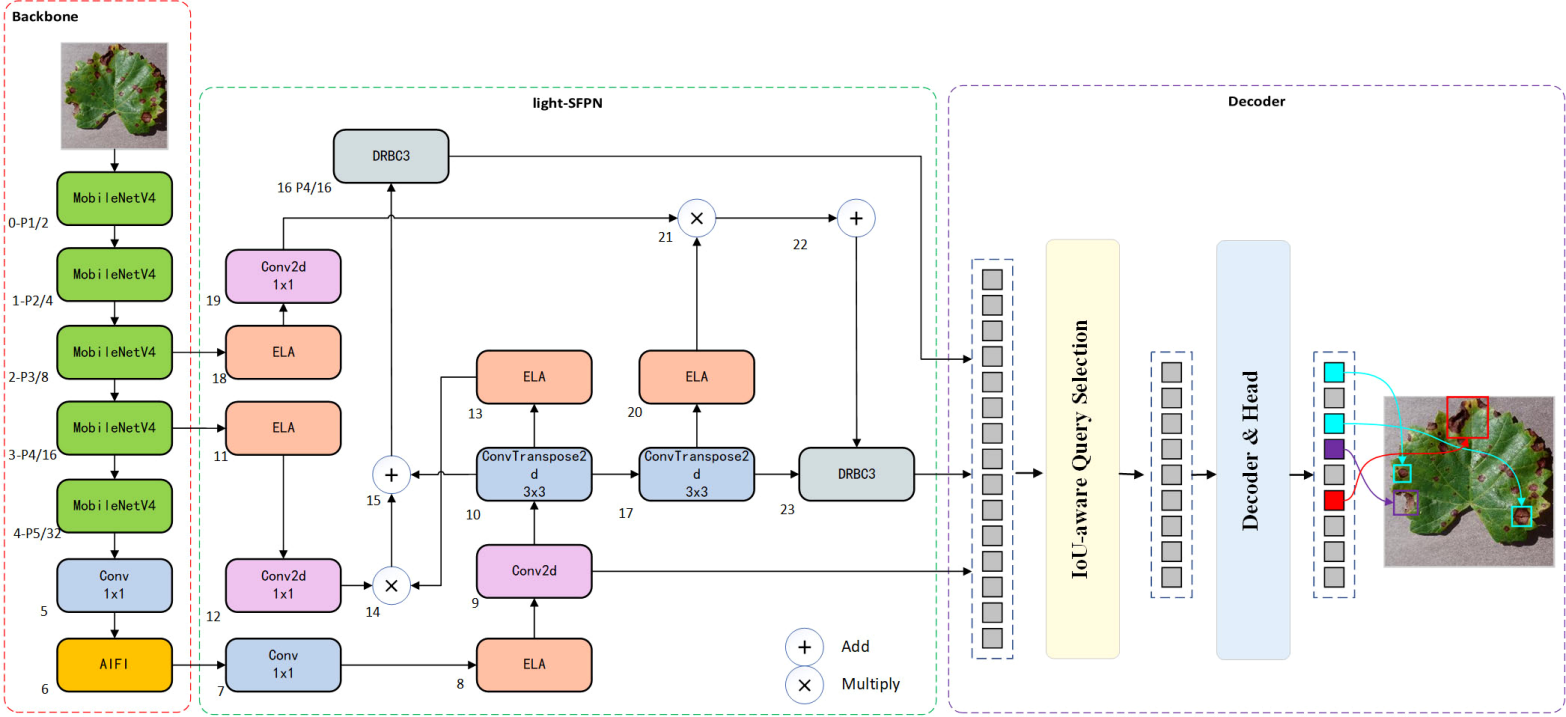
Improved RT-DETR network structure diagram(MHDI-DETR).

As target detection evolves, RT-DETR, a variant of DETR Zhu et al. (2020), addresses the limitations of real-time detection when compared to other models such as SSD Liu et al. (2016) and FLOPs Jiang et al. (2022), maintaining both accuracy and detection speed. Additionally, achieving a lightweight grape leaf disease detection model under natural conditions with limited resources is challenging. Thus, this study proposes the MHDI-DETR architecture to optimally address these challenges. In this paper, MobileNetv4 is employed as the backbone network, utilizing Universal Inverted Bottleneck Blocks (UIB) Qin et al. (2024), which significantly improves computational efficiency and operational speed. The proposed DRBC3 structure is created by fusing the DRB Ding et al. (2024) with the RepC3 module, playing a crucial role in the final stage of feeding the detection head. It uses a combination of dilated and normal convolutions to obtain a more comprehensive feature map by utilizing a larger receptive field while minimizing parameter size. The proposed light-SFPN structure integrates the Hierarchical Scale Feature Pyramid Network(HSFPN) structure from Deformable Detr Chen et al. (2024) with the improved DRBC3 module and ELA Xu and Wan (2024) design, aiming to reduce computational cost and inference latency while ensuring recognition efficiency for small targets. By integrating GIoU Rezatofighi et al. (2019) and Focaler-IoU Zhang and Zhang (2024) into Focaler-GIoU, the model becomes more focused on difficult samples, enhancing regression efficiency. A thorough evaluation shows that the MHDI-DETR model surpasses the baseline RT-DETR and other mainstream detection algorithms in balancing a lightweight design and accuracy. **Figure 3** presents the overall structure of the MHDI-DETR model.

### 2.5 Backbone network improvements

**Figure 4:**
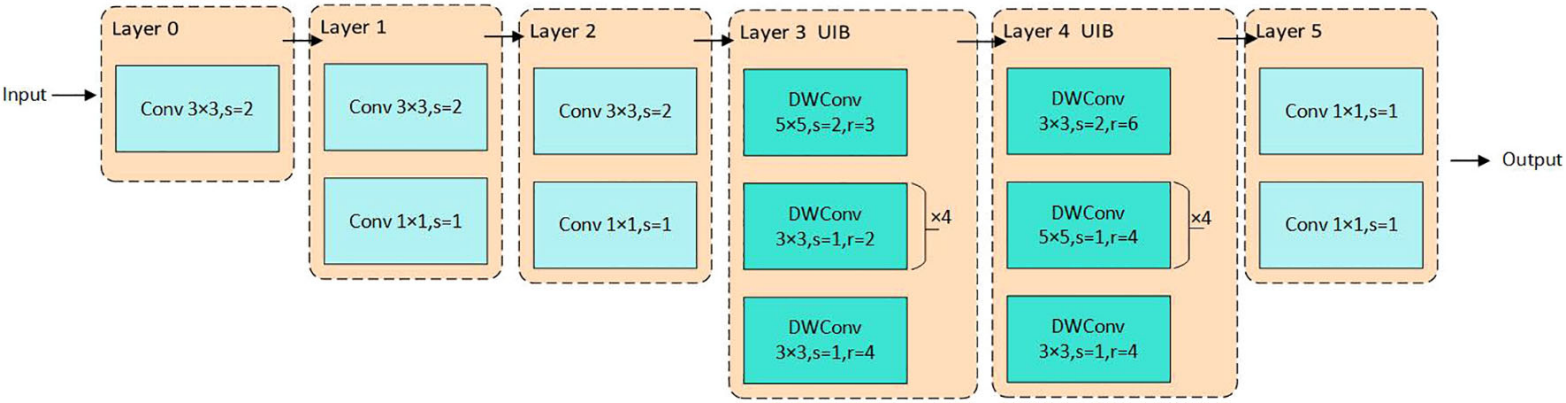
Improved lightweight backbone network diagram.

The original ResNet-18 network backbone is unsuitable for deployment on mobile devices with limited storage due to its deeper network structure and higher number of parameters, leading to increased computational complexity and larger models. Thus, selecting a feature extraction backbone suitable for resource-constrained devices is crucial. The ResNet-18 backbone is replaced with MobileNetv4, utilizing its advanced UIB module alongside ordinary convolution to provide more efficient spatial and channel mixing capabilities. **Figure 4** illustrates the backbone network utilized for MHDI-DETR. The first three layers apply 3 × 3 convolutions with a step size of 2 for feature extraction, where Layer1 and Layer2 are paired with a 1 × 1 Pointwise Convolution to fuse information from different channels. Deeper features are then extracted through the UIB, while 3 × 3 and 5 × 5 DWConv are employed in Layer3 and Layer4, respectively, to maintain a rich capture of spatial features while progressively reducing the feature map resolution. In Layer3, the 5 × 5 DWConv captures larger spatial features, which are further refined by four consecutive 3 × 3 DWConvs with a step size of 1 and a dilation rate of 2. In Layer4, a 3 × 3 DWConv is initially used for feature extraction and downsampling, followed by another 3 × 3 DWConv to capture more global information without altering the resolution. Finally, Layer5 outputs the final result for channel count adjustment. This module simplifies network design, enables network structure adjustments at different stages, and facilitates its application to grape leaf disease detection tasks. The corresponding mathematical formula is expressed as follows Qin et al. (2024).


(1) P(X)=X∗K+b

(2) $$D\left(X\right)=\;\sum_{g=1}^{G}X_{g}*\;D_{g}+b$$

(3) E(X)=P(X)⊗W 

In Equation 1, assuming the input feature map is *X*, where ∗ represents the convolution operation, *K* is the convolution kernel, and *b* is the bias term, the Pointwise Convolution is denoted as *P*(*X*). In Equation 2, *G* represents the number of input channel groupings, and *D_g_* is the DWConv of the *g*th group. The Deep Convolution operation is then expressed as *D*(*X*). In Equation 3, *E*(*X*) increases the number of channels through Pointwise Convolution, providing additional feature representations for subsequent operations. Here, ⊗ denotes matrix multiplication, and *W* represents the weight matrix.

### 2.6 Applying dilated convolutional reparameterisation blocks

**Figure 5:**
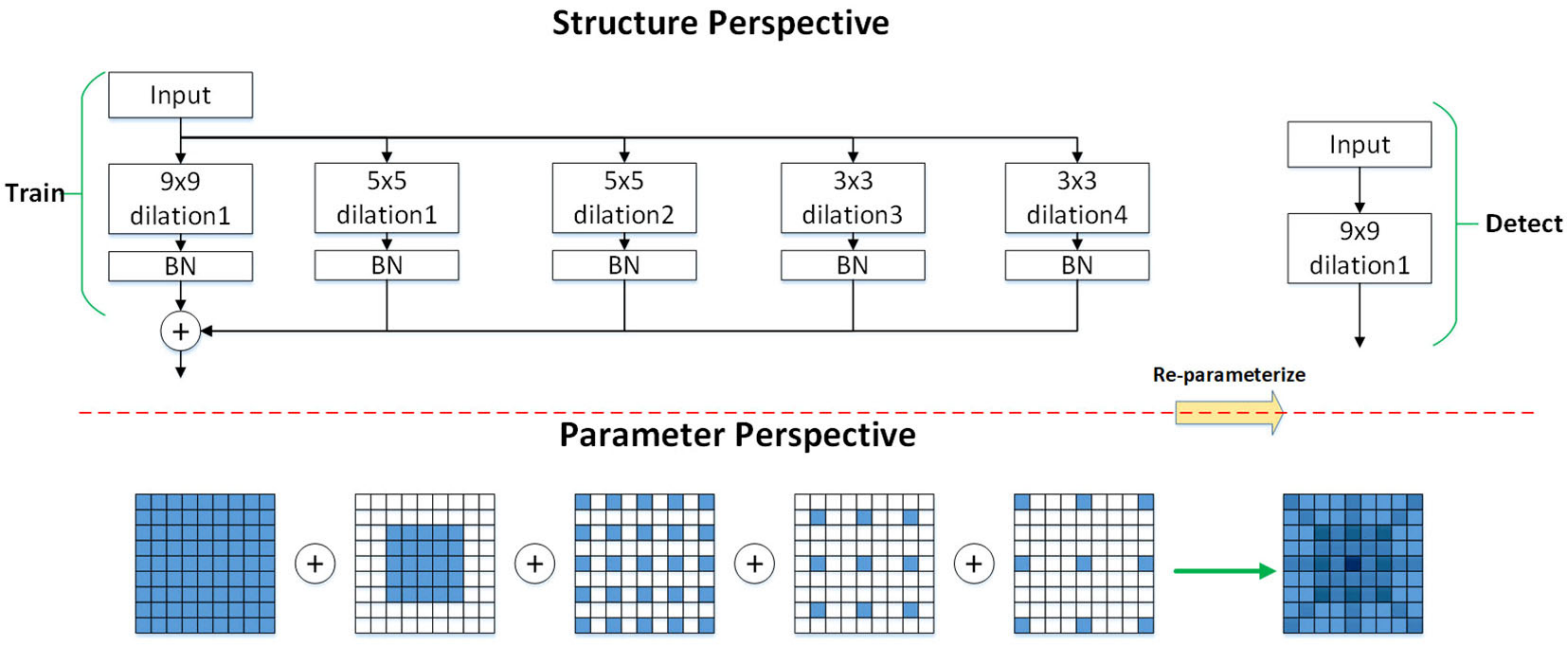
The DRBC3 module.’Train’ represents the training phase, and ‘Detect’ represents the inference phase. The numbers following ‘dilation’ indicate different dilation rates. By employing various dilation rates, the model can capture features at different scales. For instance, convolutional kernels with dilation rates of 1, 2, and 3 capture local, intermediate, and long-range pixel relationships, respectively.


It has been observed that using multiple branches during model training accelerates the process, while employing a single branch during inference reduces inference time Ding et al. (2021). Therefore, the DilatedReparamBlock structure is fused with the RepC3 module in the original neck network, forming the DRBC3 module, which applies convolutions of the same size to achieve a larger receptive field with reduced computational effort, thereby enhancing meaningful feature extraction. Additionally, since dilated convolution adjusts the receptive field size based on the dilation rate, it captures a wider region of the input feature map without increasing the number of parameters, allowing the model to focus on important local regions. As illustrated in **Figure 5**, representing the DilatedReparamBlock structure, during the training phase, the input feature map enters the DRBC3 module, undergoing multiple convolution operations with varying kernel sizes and dilation rates to process the input maps in parallel. The outputs of each convolution are processed by BN and ReLU6 activation functions, and the feature maps are summed element-wise to fuse into a final feature map. Convolution kernels with varying dilation rates extract different levels of information, and the element-wise summation fuses this information, allowing the model to utilize these features simultaneously, enhancing feature representation and ensuring that all convolution outputs maintain consistent spatial dimensions. During the detection phase, the scale information carried by each branch is fused using the reparameterization technique, significantly speeding up inference and reducing model size. This technique simplifies the convolution operations, improving computational efficiency during inference. The output of the DRBC3 module is expressed as shown in Equation 4, Ding et al. (2024).

(4) $$y=BN(ReLU6\;\left(BN\;\left(W*x\right)\right)+\sum_{i=1}^{n}ReLU6\;(BN\;(W_{dilated,i}*x)))$$

(5) $$k_{equivalent}=(k-1)\;r+1$$

(6) $$W^{\prime }=convtranspose2d\;(W,I,stride=r)$$

Where *x* represents the input, *y* represents the output, *BN* stands for Batch Normalization, and ∗ denotes convolution. *W* represents the standard convolutional weights, and *W* ∗ *x* denotes the convolution operation on the input feature maps using a standard convolution kernel to capture local features, which are subsequently processed by *BN* and *ReLU*6 activation functions. *W_dilated,i_* represents the *i*th dilated convolution kernel, and *W_dilated,i_* ∗ *x* denotes the convolution of the input feature maps using the dilated convolution kernel, which expands the receptive field with varying dilation rates, followed by *BN* and *ReLU*6 activation functions. The output feature maps from different convolution operations are summed element-wise, fusing the multi-scale, multi-receptive field features. A uniform batch normalization is performed on the fused final feature maps for the *BN* layer. To reduce computational overhead during inference, a structural reparameterization method is employed to equivalently convert multiple dilated convolution kernels into a single large non-dilated convolution kernel. The equivalent kernel size for the dilated convolution is expressed in Equation 5, where *k* represents the kernel size and *r* denotes the expansion rate. The equivalent transformation is shown in Equation 6, where *W* denotes the dilation convolution and *I* represents the unit convolution kernel of size 1 × 1. Substituting the RepC3 module with the DRBC3 module boosts feature extraction and enhances model performance while preserving computational efficiency.

### 2.7 Improved neck feature fusion network

Although using the MobileNetv4 feature extraction backbone significantly reduces model size and improves performance while maintaining efficiency, there remains substantial room for improvement in terms of computational cost and the number of parameters for the CNN-based Cross-scale Feature-fusion Module (CCFM). Furthermore, the CCFM is unable to prioritize features from different layers, despite its ability to fuse them. Thus, we incorporate the HS-FPN structure from Chen et al. (2024), the ELA concept proposed in Xu and Wan (2024), and the idea of dilated convolutional reparameterization from Ding et al. (2024) to design the light-SFPN module. This module compensates for the CCFM’s loss of small target information during convolution and downsampling by employing a more flexible feature fusion approach. This approach allows the model to adaptively select features from different layers for fusion, enabling it to focus on regions of interest and improve recognition accuracy. The structure consists of a Feature Selection Module (FSM) and a Feature Fusion Module (FFM). It was found that compared to the Channel Attention (CA) used in the original HS-FPN, Efficient Local Attention (ELA) improves computational efficiency without sacrificing performance. Furthermore, unlike CA, ELA does not require dimensionality reduction, allowing it to maintain the channel dimensionality of the input feature map, thereby retaining more feature information.

#### 2.7.1 Efficient local attention module

As illustrated in **Supplementary Figure S1** of the **Supplementary Materials**, the ELA module specifically receives features from the previous layer and applies Adaptive Average Pooling to compress the feature map along both the height and width directions. Strip Pooling along the height direction generates an average for each horizontal position, producing a feature map that contains horizontal coordinate information, as shown in Equation 7, where *z_h_* (*h*) represents the global information embedding of the input feature map *x* in the height dimension. Similarly, Strip Pooling along the width direction generates an average for each vertical position, forming a feature map containing vertical coordinate information, as shown in Equation 8, where *z_w_* (*w*) represents the global information embedding of the input feature map *x* across the width. This process provides the necessary spatial context for the subsequent 1D convolution. Next, these coordinate features are processed by 1D convolution to enhance position-specific feature responses and generate preliminary attention features for the attention module. The coordinate feature maps processed through 1D convolution and group normalization are used to generate the final attention weights *y_w_* and *y_h_*. *σ* represents the Sigmoid function. As shown in Equations 9, 10, these weights guide the network to focus on important feature regions.


(7) $$z_{h}\left(h\right)=\frac{1}{H}\sum_{i=0}^{H-1}x\;(h,i)$$

(8) $$z_{w}\left(w\right)=\;\frac{1}{W}\sum_{j=0}^{W-1}x\;(j,\;w)$$

(9) $$y_{h}=\sigma \left(GN\;(Conv1D\;(z_{h}))\right)$$

(10) $$y_{w}=\sigma \left(GN\;(Conv1D\;(z_{w}))\right)$$

#### 2.7.2 Light-SFPN feature fusion architecture

The FSM employs GAP (Global Average Pooling) to obtain the processed feature maps *f_avg_* and GMP (Global Max Pooling) to derive the feature maps *f_max_*, as shown in Equations 11, 12.


(11) $$f_{avg}=GAP\;(x)$$

(12) $$f_{\text{max}}=GMP\;(x)$$

Next, the weights of each channel are calculated using the Sigmoid activation function, as shown in Equation 13, where *w* represents the weight, *FC* denotes the fully connected layer, and *σ* represents the Sigmoid activation function.


(13) $$w=\sigma \left(FC\;(f_{avg})+FC\;(f_{max})\right)$$

The weight information is subsequently dot-multiplied with the feature map to generate a filtered feature map, as shown in Equation 14.


(14) $$f_{filtered}=x⊗w$$

**Figure 6:**
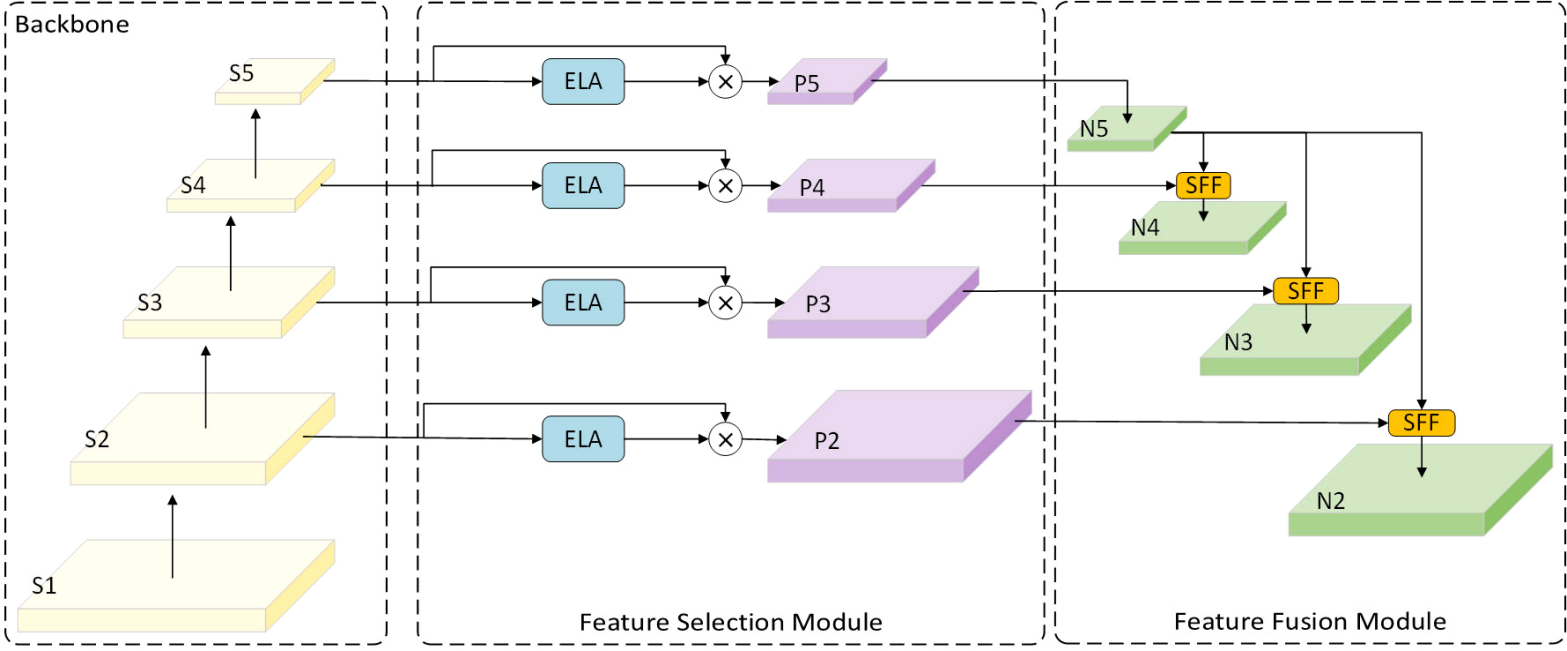
HS-FPN-ELA structure.

The FSM calculates the global average pooling and maximum pooling of the feature maps to obtain the importance weights for each channel, and then applies these weights to the original feature maps to select the most important feature information. Next, the weighted feature maps (p2–p5) filtered by ELA are input into the FFM, which is used to fuse high-level features (rich in semantic information but with imprecise localization) with low-level features (with precise localization but limited semantic information). **Figure 6**, Chen et al. (2024) illustrates this process.

**Figure 7:**
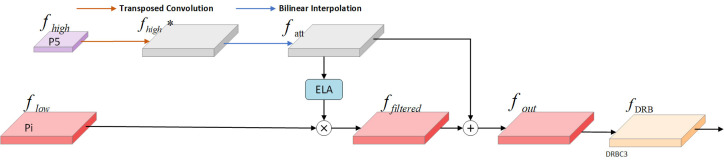
SFF module in light-SFPN.

The SFF module is a feature fusion technique designed to enhance model performance by integrating high-level semantic information with low-level detail information. The functioning of the SFF module can be outlined in the following steps: First, the high-level feature maps are resized to match the resolution of the low-level feature maps using transposed convolution and bilinear interpolation operations. Next, the high-level feature maps are used as attention weights to guide the model’s focus toward important regions of the low-level feature maps, enabling the model to selectively emphasize areas in the low-level features that align with the high-level features. Finally, using these attention weights, the semantic information of the high-level features is combined with the detailed information of the low-level features. This fusion not only preserves the rich semantics of high-level features but also incorporates the fine texture and edge details of low-level features. **Figure 7** illustrates the SFF module within the light-SFPN architecture.

Given an input high-level feature *f_high_* ∈ ℝ*^C^* ^×^ *^H^* ^×^ *^W^* and an input low-scale feature $f_{low}\in {\mathbb{R}}^{C\times H_{1}\times W_{1}}$ , the high-level feature is initially convolved with a step size of 2 and a kernel size of 3×3. The transposed convolution is then extended to obtain $f_{high^{*}}\in {\mathbb{R}}^{C\times 2H\times 2W}$ . Next, to align the resolution and dimensionality of the high-level and low-scale features, the high-level features are adjusted using Bilinear Interpolation, yielding $f_{att}\in {\mathbb{R}}^{C\times H_{1}\times W_{1}}$ . This process is demonstrated in Equation 15, Chen et al. (2024).


(15) $$f_{att}=BilinearInterpolation\;\left(TransposedConvolution\;\left(f_{high}\right)\right)$$

Next, the high-level features are converted into the corresponding attention weights *y_h_* and *y_w_* using the ELA module to filter the low-scale features and obtain features with the same dimensionality. This process is illustrated in Equation 16.


(16) $$f_{filtered}=f_{low}⊗y_{h}⊗y_{w}$$

Second, the filtered low-scale features *f_filtered_* are fused with *f_att_* to obtain $f_{out}\in {\mathbb{R}}^{C\times H_{1}\times W_{1}}$ , as shown in Equation 17.


(17) $$f_{out}=f_{filtered}+f_{att}$$

Finally, *f_out_* is processed through the DRBC3 module to produce the final feature map *f_DRB_*, as shown in Equation 18.


(18) $$f_{DRB}=DRBC3\left(f_{out}\right)$$

### 2.8 Improved loss function

The RT-DETR model utilizes GIoU to compute the bounding box regression loss. While it addresses the issue of IoU’s gradient vanishing when the two bounding boxes do not overlap, it may converge more slowly when there is an imbalance between simple and difficult samples, particularly when there is a significant difference in target sizes and shapes (e.g., small targets often represent difficult samples). Focaler-IoU addresses this issue by dynamically adjusting parameters, allowing the model to focus on either difficult or simple samples, thereby improving detection performance for specific sample types. Specifically, IoU loss is reconstructed through linear interval mapping to focus on different regression samples across various detection tasks. Combining these techniques preserves GIoU’s localization accuracy while enabling the model to focus on small target samples, fully leveraging the strengths of both approaches. GIoU resolves the issue of IoU’s gradient vanishing in cases of no overlap by introducing a minimum enclosing frame that contains both the predicted and ground-truth boxes, thereby improving localization performance in target detection. The computation of GIoU and its loss is shown in Equations 19, 20.


(19) $$GIoU=IoU-\frac{\left|C\;(A\cup B)\right|}{\left|C\right|}$$

(20) $$L_{GIoU}=1-GIoU$$

(21) $$IoU=\frac{\left(A\cap B\right)}{\left(A\cup B\right)}$$

*A* and *B* represent the predicted boxes and the ground-truth boxes, respectively, while *C* represents the smallest enclosing boxes containing both *A* and *B*, and |·| denotes the area of the region. The computation of the standard IoU, as shown in Equation 19, is detailed in Equation 21.


(22) $$IoU_{Focaler}=\{\begin{array}{cc} 0, & \text{IoU}<\text{d}\\ \frac{\text{IoU}-\text{d}}{u-d}, & d\leq \text{IoU}\leq u\\ 1, & \text{IoU}<u \end{array}$$

(23) $$L_{Focaler-GIoU}=L_{GIoU}+IoU-IoU_{Focaler}$$

To further enhance detection performance, the Focaler-GIoU loss is introduced. Focaler-GIoU reconstructs the IoU through linear interval mapping, allowing it to better focus on samples of varying difficulty. As shown in Equation 22, *d* and *u* are tuning parameters that control the range of samples to focus on. The Focaler-GIoU loss is then calculated as shown in Equation 23.

### 2.9 Evaluation indicators

Model performance improvements are comprehensively evaluated based on three key metrics: precision, computational complexity, and processing speed. Precision is evaluated using precision rate (P), recall rate (R), and the mean average precision (mAP) across all categories. Computational complexity is assessed by floating-point operations (FLOP) and parameter counts (Parameters), and processing speed is measured by frames per second (FPS), representing the model’s inference speed, with FPS computed based on the validation test set.

The accuracy metric evaluates the performance of the model’s detection, where a true positive (TP) refers to the correct identification of a positive case, a false positive (FP) refers to an incorrect judgment of a positive case, a true negative (TN) refers to the correct identification of a negative case, and a false negative (FN) refers to an incorrect omission of a positive case. The accuracy formula is provided in Equation 24. The following equations present the formulas for AP and mAP, where higher values of both metrics indicate better algorithm performance. P denotes the precision rate, R denotes the recall rate.


(24) $$precision=\;\frac{TP}{TP+FP}$$

Recall represents the proportion of actual targets that are correctly detected by the model. A high recall rate indicates that the model successfully identifies most positive samples and has a reduced likelihood of missing detections. The formula for recall is provided in Equation 25.


(25) $$recall=\;\frac{TP}{TP+FN}$$

AP is a composite metric used to evaluate the performance of a target detection model by measuring its ability to detect objects across different thresholds. mAP offers a composite metric for evaluating the model’s detection performance across all categories. A higher mAP indicates better model performance in detecting all categories across various thresholds. mAP integrates precision and recall, offering a more complete assessment of the model’s performance. The mAP calculation is shown in Equations 26, 27.


(26) $$AP=\int_{0}^{1}P\;\left(R\right)dR$$

(27) $$mAP=\;\frac{1}{N}\sum_{j=1}^{n}AP_{j}$$

The model parameter count refers to the total number of trainable parameters in the model. The number of parameters directly impacts the model’s storage requirements and training time. Computational power, typically expressed in floating-point operations (FLOPs), measures the amount of computation required for one forward propagation. Models with high computational power experience slower inference and increased energy consumption, which not only affects real-time application performance but also demands significant resources. FPS measures the processing speed of a model in real applications, indicating the number of frames per second that can be handled. Higher FPS indicates better real-time performance of the model in practical applications. The formula for FPS is provided in Equation 28, where *t_avg_* represents the average time taken by the model to process a single frame.


(28) $$FPS=\;\frac{1}{t_{avg}}$$

## 3 Results and analysis

### 3.1 Experimental configuration

The training of the experimental model was conducted on a cloud server. The configuration of the experimental environment is provided in **Supplementary Table S3** of the **Supplementary Materials**. The model’s hyperparameters are configured as follows: a preset image size of 640 × 640, 150 training iterations, a batch size of 8, and 4 working threads for data loading. The initial learning rate is set to 0.0001, the final learning rate is adjusted to 1, and there are 2000 warm-up iterations. The AdamW optimizer is utilized. All other training hyperparameters are maintained at their default values unless otherwise specified.

### 3.2 Ablation experiment

#### 3.2.1 Ablation experiments on light-SFPN structures

The ablation experiments presented in **Table 1** demonstrate that using different neck network configurations allows for a clear observation of the effects of various network structures on model performance. Compared to the original model, the P and mAP metrics improve with the use of HSFPN, while the number of parameters and computational requirements are reduced to varying degrees. The HS-FPN+ELA strategy significantly enhances mAP, albeit with moderate increases in computational complexity and parameter count, both of which remain within an acceptable range. The use of light-SFPN further reduces the model size while maintaining accuracy and appropriately increases inference speed, leading to improvements in FPS. The optimized model performance enhances the efficient utilization of computational resources.

**Table 1:** Comparison of ablation experiments of light-SFPN.

Model	P (%)	mAP50 (%)	mAP50:95 (%)	Parameters (M)	GFLOPs	FPS
RT-DETR	95	91.4	71.3	19.87	57	57.7
RT-DETR+HS-FPN	96.4	91.5	71.6	18.11	53.3	63.4
RT-DETR+HSFPN+ELA	96.5	92.2	72.4	18.26	53.4	63
RT-DETR+light-SFPN	96.2	92.3	72.5	17.39	46.5	67.4

#### 3.2.2 Integral ablation experiments

To thoroughly evaluate the impact of the improvement modules on the model’s performance, we carried out eight ablation studies with progressive modifications to the baseline model, RT-DETR. First, the backbone network architecture was replaced with the MobileNetv4 network featuring the UIB module. Second, the dilated convolutional reparameterized module was integrated into the RepC3 module, forming the DRBC3 module. Next, the light-SFPN structure was designed to optimize the feature fusion network. Finally, the GIoU loss function was substituted with the Focaler-GIoU loss function. Each enhancement was implemented step-by-step, and the results are summarized in **Table 2**.

**Table 2:** Performance evaluation of ablation experiments.

Experiment	MobileNetv4.Backbone	DRBC3	light-SFPN	Focaler-GloU	P(%)	mAP50(%)	mAP50:95(%)	Params(M)	GFLOPs	FPS
1.baseline	–	–	–	–	95	91.4	71.3	19.87	57	57.7
2	✓	–	–	–	97.1	91.6	72	11.31	39.5	62.5
3	–	✓	–	–	96.5	91.6	71.9	18.14	48.3	61.9
4	–	–	✓	–	97.1	92.3	72.4	17.39	46.5	62
5	–	–	–	✓	96.3	92	72.2	19.87	57	59
6	✓	✓	–	–	96.3	92.2	72.2	9.58	30.8	62.2
7	✓	✓	✓	–	96.2	92.3	72.3	8.76	29	67.4
8.ours	✓	✓	✓	✓	96.9	92.6	72.5	8.76	29	69.8

The symbol "✓" indicates that the corresponding improvement or modification was implemented in the ablation experiment.

The ablation experiment results are presented in **Table 2**. After replacing the backbone network with the MobileNetv4 network and UIB module, the evaluation metrics of P, mAP50, mAP50:95, and FPS improved by 2.1%, 0.2%, 0.7%, and 8.3%, respectively. This demonstrates that this network not only enhances detection accuracy but also accelerates detection speed, while reducing the number of parameters and computation by 43% and 30.7%, respectively. Compared to the RT-DETR model, the number of parameters and computational load decreased, while P, mAP50, and mAP50:95 increased to varying degrees following the introduction of the DRBC3 module. This indicates that the DRBC3 module outperforms the original RepC3 module in both accuracy and model size. The application of the lightSFPN structure to the neck network led to significant improvements in P, mAP50, and mAP50:95 by 2.1%, 0.9%, and 1.1%, respectively, while preserving stability across other metrics. This suggests that light-SFPN effectively enhances the fusion of multi-layer feature map information. Finally, adopting the Focaler-GIoU loss function resulted in an increase of 1.3%, 0.6%, and 0.9% in P, mAP50, and mAP50:95, respectively. Comparing Experiment 1 and Experiment 6, the combination of the MobileNetv4 network and DRBC3 module significantly reduced the number of parameters by 51.7% and computation by 46%, while maintaining efficient recognition accuracy suitable for deployment on resource-constrained devices. From Experiments 1, 6, and 7, it can be concluded that after applying light-SFPN in Experiment 7, the model’s parameter count decreased by 8.6%, GFLOPs decreased by 5.8%, and FPS improved by 8.3%, compared to Experiment 6, despite no significant increase in average accuracy. Finally, comparing Experiment 1 and Experiment 8, the results show that the proposed MHDI-DETR model outperforms the original model in several metrics, including P, mAP50, mAP50:95, and FPS, with improvements of 1.9%, 1.2%, 1.2%, and 21%, respectively. Additionally, the model’s size and computational load were significantly reduced by 56% and 49%, confirming the effectiveness of each module’s improvement in this comprehensive ablation experiment.

### 3.3 Comparison experiment

#### 3.3.1 Backbone network comparison experiment

The backbone network of the RT-DETR model utilizes four BasicBlock modules for feature extraction. A primary challenge in grape leaf disease detection is ensuring the model’s performance on resourceconstrained devices, while maintaining accuracy despite a reduced model size. To verify the performance enhancement provided by the MobileNetv4 network, several top convolutional networks were chosen for benchmarking, as illustrated in **Table 3**.

**Table 3:** Comparison of experimental results for different backbone networks.

Backbone network	P (%)	mAP50 (%)	mAP50:95 (%)	Params (M)	GFLOPs	FPS
BasicBlock	95	91.4	71.3	19.87	57	57.7
BasicBlock_Conv3XC	96.4	91.6	71.1	23.85	68.3	54.4
BasicBlock_DualConv	96.4	91.6	71.1	15.87	47.3	60
EfficientViT	96.2	91.8	71.7	10.71	27.2	32.3
BasicBlock_iRMB	96.2	91.8	71.8	16.41	49.1	49.2
BasicBlock_PConv	95.4	92	71.2	14	42.8	62
RepViT	95.5	91.8	70.6	13.3	36.3	47.8
MobileNetv4	97.1	91.6	72	11.31	39.5	62.5

As shown in **Table 3**, compared to RT-DETR, using MobileNetv4 as the backbone network significantly reduces the number of parameters and computational cost by 43% and 30.7%, respectively. Additionally, MobileNetv4 provides considerable improvements in P, mAP, and FPS. EfficientViT Liu et al. (2023), RepViT Wang et al. (2024), and MobileNetv4 all modify the backbone of ResNet-18 as lightweight networks. While EfficientViT and RepViT offer significant advantages in reducing the number of parameters and computational load, their FPS is substantially lower than MobileNetv4’s, which may be attributed to their use of Transformer architecture. This slowdown is likely due to the addition of Transformer architecture, which increases the number of computational layers and delays inference speed. Other backbone networks, such as Con3XC Wan et al. (2024), achieve balanced performance when combined with Residual Blocks. However, its computational load of 68.3 is significantly higher than other models, making it unsuitable for deployment on resource-constrained devices. DualConv Zhong et al. (2022) also delivers balanced performance, but its FPS is substantially lower than MobileNetv4, which achieves the highest FPS. Although IRMB Zhang et al. (2023), PConv Chen et al. (2023), and other mainstream lightweight convolutional networks combined with residual blocks offer some improvements over the base model, their enhancements are far less significant than those of MobileNetv4. Thus, after comparing with other mainstream lightweight convolutional networks, the UIB module used by the MobileNetv4 network demonstrates superior performance.

#### 3.3.2 Average accuracy comparison

To clearly demonstrate the effectiveness of the proposed MHDI-DETR model, average accuracy curves of mAP50 and mAP50:95 for RT-DETR and MHDI-DETR were plotted, with blue representing the RT-DETR model and orange representing the MHDI-DETR model, as illustrated in **Supplementary Figure S2** of the **Supplementary Materials**. The MHDI-DETR model consistently outperformed the RT-DETR model after approximately 20 training rounds until completion. This finding indicates that, despite the reduction in size of the MHDIDETR model by half compared to the RT-DETR model, it still achieves excellent performance in detecting grape leaf diseases.

#### 3.3.3 Model loss comparison

**Figure 8:**
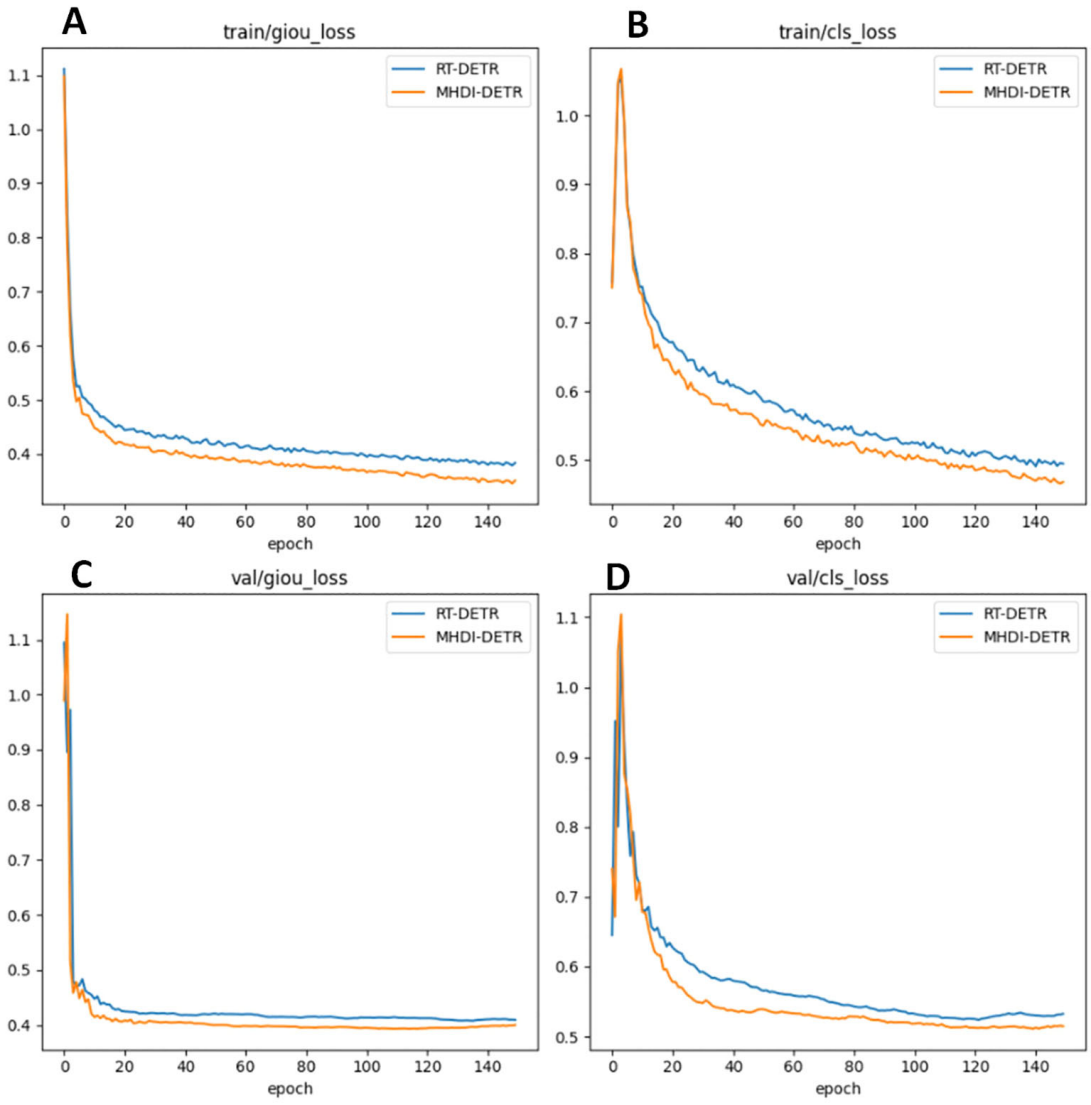
Comparison of loss curves for the RT-DETR model and MHDI-DETR model across different loss types. **(A)** IoU loss during training, **(B)** Classification loss during training, **(C)** IoU loss during validation, **(D)** Classification loss during validation. In this comparison, the RT-DETR model utilizes the GIoU loss function, while the MHDI-DETR model employs the Focaler-GIoU loss function.

To verify the advantages of the MHDI-DETR model over the RT-DETR model, a comparison of the loss curves was performed, and the visual results are presented in **Figure 8** The figure displays the IoU and classification loss curves for both the RT-DETR and MHDI-DETR models on the training and validation sets. In the figure, the blue line represents the RT-DETR model, while the orange line represents the MHDI-DETR model. The MHDI-DETR model quickly reduces both IoU and classification losses early in the training process and maintains low loss values throughout. Furthermore, the loss curves on the validation set indicate that the MHDI-DETR model exhibits better generalization, maintaining low loss on unseen data. Overall, the MHDI-DETR model demonstrates higher accuracy and stability in target localization, classification, and regression tasks. These experimental results clearly highlight the superiority of the MHDI-DETR model in leaf disease detection tasks.

#### 3.3.4 Comparison of model visualisation

**Figure 9:**
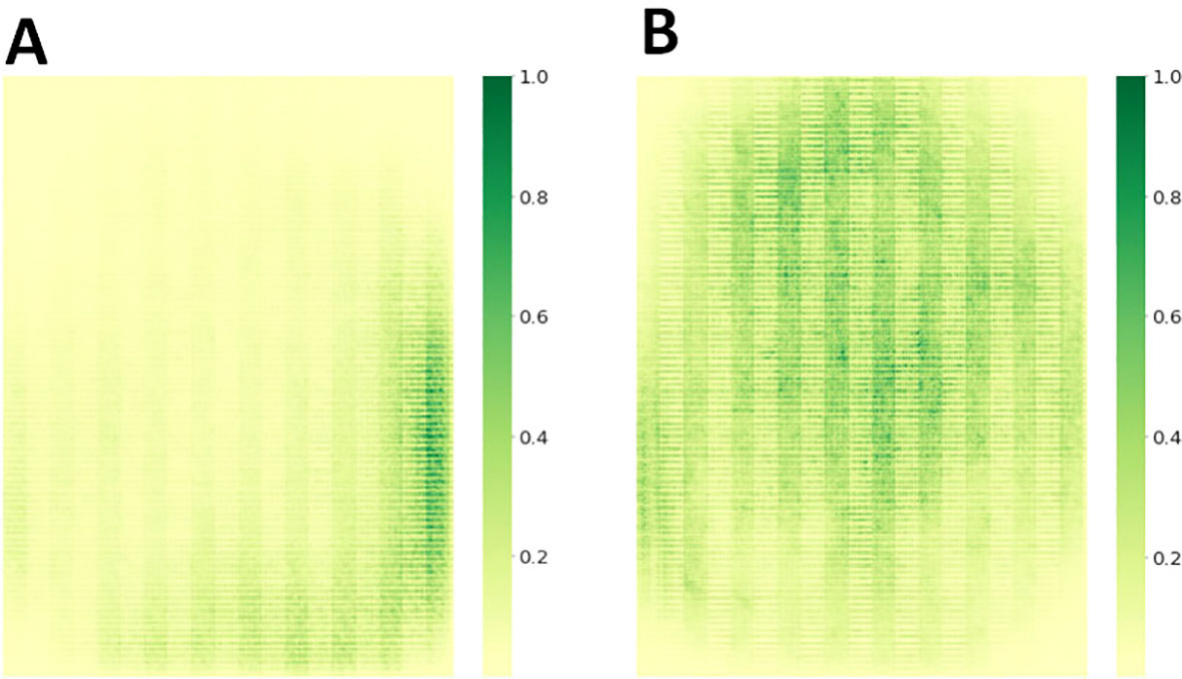
Comparison of receptive field sizes. **(A)** Receptive field of the MobileNetv4 backbone without the DRBC3 module. **(B)** Receptive field of the MobileNetv4 backbone with the DRBC3 module incorporated.

To highlight the advantages of the MHDI-DETR model more clearly and intuitively, several sets of visualisation experiments were conducted to fully assess its benefits across various aspects. First, a comparison of the receptive fields was conducted. In target detection tasks, where the size of the model’s receptive field and feature extraction capability are critical to performance, the receptive fields of the improved models were analysed and compared using gradient visualisation techniques. The receptive fields were visualised before and after applying the DRBC3 module, as shown in **Figure 9**, demonstrating the size and distribution of the receptive fields. The receptive fields of the MHDI-DETR model are more homogeneous and cover critical regions more effectively, improving detection accuracy and model robustness. A comparison of the receptive fields reveals that the MHDI-DETR model excels in feature extraction, offering a larger and more optimally distributed receptive field. These improvements allow the model to capture key information from the input image more effectively, thereby enhancing overall performance.

**Figure 10:**
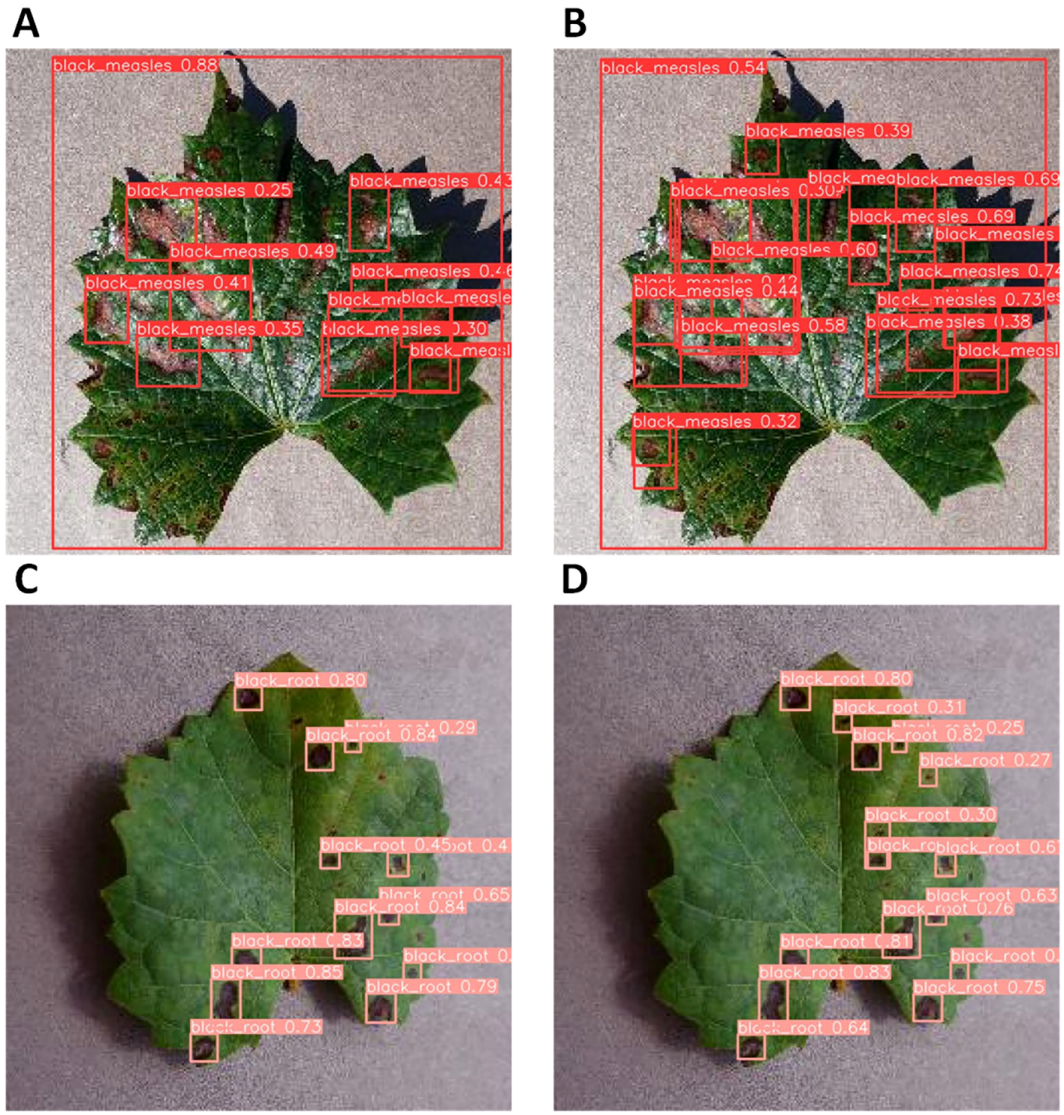
Comparison of inference performance between MHDI-DETR and RT-DETR. **(A, B)** show the model’s detection performance before and after improvement. on black measles disease, respectively.**(C, D)** show the detection performance of the model before and after improvement on black rot disease, respectively.

Secondly, to evaluate the detection of small, hard-to-detect target diseases, we tested the effectiveness of RT-DETR and MHDI-DETR by conducting comparison experiments on two selected diseases. By comparing the detection results before and after the improvements, the effectiveness and superiority of the improved method can be visually observed. **Figure 10** presents the detection results for two diseases, black measles and black rot. In A, some instances of black measles were detected, but issues of missed detection and low confidence were observed. In contrast, all black measles were accurately detected in B, with significantly higher confidence and almost no missed detections. In C, smaller and more unevenly distributed black rot targets increased detection difficulty, resulting in many small targets going unrecognized, which affected the model’s accuracy. In D, black rot was detected accurately, with no obvious omissions or misdetections, and the detection results were reliable. This confirms that the model achieves superior results in detecting small targets.

**Figure 11:**
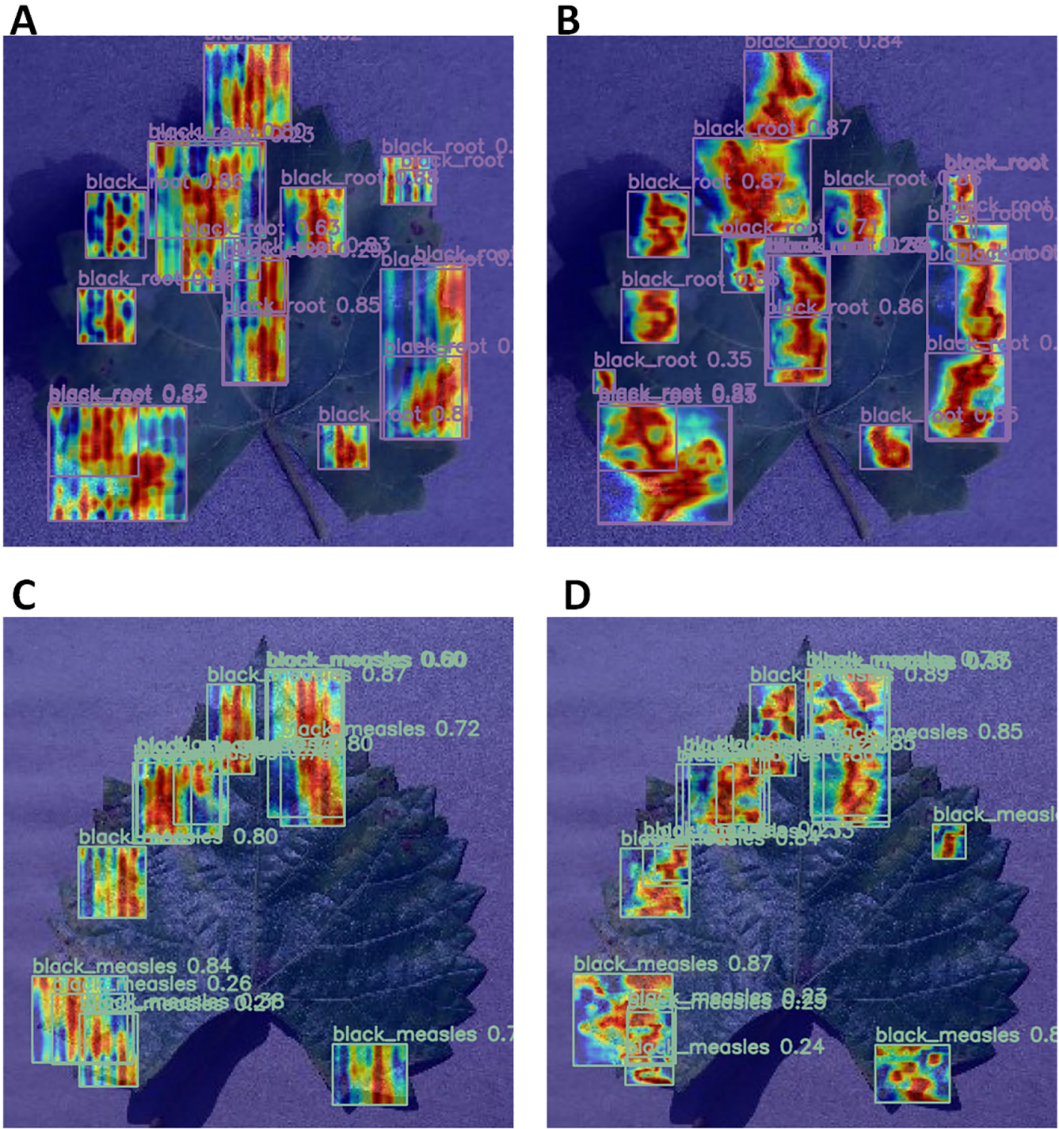
Comparison of Heatmaps for RT-DETR and MHDI-DETR Models in detecting black rot and black measles diseases. **(A)** Heatmap of black rot disease using the RT-DETR model. **(B)** Heatmap of black rot disease using the MHDI-DETR model. **(C)** Heatmap of black measles disease using the RT-DETR model. **(D)** Heatmap of black measles disease using the MHDI-DETR model.

Finally, a comparison of the heat maps generated by the two models was conducted. **Figure 11** depicts the models’ focus and accuracy in identifying diseased areas. Heat maps for both models were generated using GradCAMPlusPlus to illustrate their focus on detecting diseased areas. By analyzing the coverage and concentration of the heat maps, their ability to focus on different regions was assessed. The heat maps in A and C appear scattered and unfocused, with several diseased regions not accurately detected. The RT-DETR model demonstrates low focus in several regions, resulting in significant false positives and omissions in detecting both black rot and black measles. The errors generated are substantial, and the detection results lack sufficient stability. In contrast, B and D exhibit greater brightness and concentration in the black rot and black measles regions, indicating higher detection confidence and more precise focus on the lesion areas. Overall, the MHDI-DETR model demonstrated superior accuracy and confidence in plant disease detection, as revealed by the comparative analysis of the heat maps.

### 3.4 Comparison of different detection models

To thoroughly assess the effectiveness of the MHDI-DETR model against mainstream detection models for grapevine leaf diseases, including black measles, black rot, blight, and healthy samples, six widely used detection models—YOLOv5, YOLOv6, YOLOv7, YOLOv8, YOLOv9, and RT-DETR-r34—were selected for comparative testing with MHDI-DETR. The dataset and experimental conditions were maintained consistently across all models. This comparison aimed to validate the effectiveness and advantages of the MHDI-DETR models under uniform dataset and experimental setups. All models were trained and evaluated using identical training parameters, optimization strategies, and hardware environments. The detailed comparison results is presented in **Table 4**.

**Table 4:** Performance metrics comparison across different models.

Model	P (%)	mAP50 (%)	mAP50:90 (%)	Parameters (M)	GFLOPs
YOLOv5	93	91.9	71.6	20.86	48
YOLOv6	93.4	91.8	72.7	18	44
YOLOv7	93.4	91.8	71.4	36	105
YOLOv8	93.6	91.9	72.2	25.8	78.7
YOLOv9	95.7	92.3	73.6	31.19	116.8
RT-DETR-r34	97	92.3	71.5	31.1	88.8
MHDI-DETR	96.3	92.6	72.9	8.76	29

The specific performance of each model across various metrics is shown in **Table 4**. Regarding P, the MHDI-DETR model achieved an accuracy of 96.3%, second only to RT-DETR-r34’s 97%, and surpassing all the YOLO series models. YOLOv9 exhibited the highest accuracy (95.7%) among the YOLO series models, while YOLOv5 had the lowest accuracy (93%). These results indicate that the MHDI-DETR model demonstrates high accuracy and reliability in disease detection tasks. Regarding average accuracy (mAP), the mAP50 of the MHDI-DETR model reached 92.6%, the highest among all models, and slightly higher than the 92.3% achieved by YOLOv9 and RT-DETR-r34. Furthermore, the MHDI-DETR model achieved the highest mAP50:90 at 72.9%, followed closely by YOLOv6 with 72.7%. These results demonstrate that the MHDI-DETR model maintains high detection accuracy across various IoU thresholds, outperforming both the YOLO series and RT-DETR series models. The number of parameters and floating-point operations (FLOPs) are critical indicators of model complexity and computational efficiency. The MHDI-DETR model contains only 8.76M parameters, significantly fewer than those of more complex models such as RT-DETR-r34 (31.1M), YOLOv7 (36M), and YOLOv9 (31.19M). While YOLOv5 and YOLOv6 have lower parameter counts of 20.86M and 18M, respectively, the MHDI-DETR model still outperforms them in overall performance. Additionally, the MHDI-DETR model has 29 GFLOPs, significantly lower than that of the YOLOv5 to YOLOv9 models, indicating reduced computational complexity and greater suitability for resource-constrained application environments. In conclusion, the improved MHDI-DETR model demonstrated significant advantages across all metrics, proving its superiority and practicality in grape leaf disease detection tasks.

## 4 Discussion

The experimental outcomes indicate that the proposed MHDI-DETR model outperforms conventional models in detecting grape leaf diseases. The effectiveness of each enhanced component, including the lightweight MobileNetv4 backbone, the DRBC3 module, the light-SFPN structure, and the Focaler-GIoU loss function, was confirmed through ablation studies. The incorporation of MobileNetv4 as the backbone network effectively reduces the model’s parameter count and computational load, while enhancing detection accuracy. Specifically, compared to RT-DETR, MobileNetv4 reduces the model’s parameters by 48% and computational load by 30.7%, while improving accuracy by 2.1 percentage points. This indicates that MobileNetv4 can extract more discriminative features while maintaining computational efficiency. The DRBC3 module enhances feature extraction by combining dilated convolution with RepC3 convolution, significantly improving detection accuracy and efficiency. Experimental results show that P and mAP50:95 improve by 1.5% and 0.6%, respectively, with the DRBC3 module, while reducing parameters and computational load. Additionally, the light-SFPN structure enhances multi-scale feature fusion, particularly for small target detection, by incorporating the efficient local attention (ELA) mechanism. Ablation experiments demonstrate that the light-SFPN structure improves mAP50 and mAP50:95 by 0.9% and 1.1%, respectively, while reducing computational complexity and parameter count. The Focaler-GIoU loss function enhances model performance by dynamically adjusting parameters to focus on difficult samples, thereby improving regression accuracy. Compared to the traditional GIoU loss function, Focaler-GIoU improves mAP50 by 0.6% and mAP50:95 by 0.9%, while converging faster. Comprehensive analysis showed that the MHDI-DETR model excelled in grape leaf disease detection, particularly for small-target diseases like black measles and black rot, with significantly higher detection accuracy, confidence. agricultural practices, with the goals of enhancing crop quality, minimizing waste, and improving overall agricultural productivity.

## 5 Conclusion

In this study, a lightweight model, MHDI-DETR, is proposed for grape leaf disease detection, achieving significant improvements across several aspects. By incorporating the MobileNetv4 backbone network, DRBC3 module, light- SFPN structure, and Focaler-GIoU loss function, the model enhances detection accuracy and efficiency while significantly reducing the number of parameters and computational load. Specifically, the MHDI-DETR model achieves a 1.2% improvement in both mAP50 and mAP50:95, while GFLOPs and the number of parameters are reduced by 56% and 49%, respectively. Experimental results confirm the validity and reliability of the MHDI-DETR model in practical applications. Future research will concentrate on incorporating the model into smart grape harvesting robots for field validation and investigating detection techniques for diseases in other fruits and agricultural products, advancing the development of smart agriculture. These initiatives are focused on promoting smart and sustainable agricultural practices, with the goals of enhancing crop quality, minimizing waste, and improving overall agricultural productivity.

## Data Availability

The original contributions presented in the study are included in the article/**Supplementary Material**. Further inquiries can be directed to the corresponding author.
