# Supplementary material for: A lightweight MHDI-DETR model for detecting grape leaf diseases
Source: Front Plant Sci. 2024 Dec 6;15:1499911. doi: 10.3389/fpls.2024.1499911 (PMC11659005; doi:10.3389/fpls.2024.1499911)
Supplement: Supplementary file 1 [file DataSheet1.pdf]

Supplementary Material

1 SUPPLEMENTARY TABLES AND FIGURES.

1.1 Tables

Table S1. Number of grape leaf disease samples.

Types	Numbers
black_measles	1368
black_rot	1129
blight	1059
healthy	995
total	4551

Table S2. Sample counts for each class in dataset.

Types	Training	Validation	Test
black_measles	1102	128	138
black_rot	900	122	107
blight	839	107	113
healthy	801	98	96
total	3642	455	454

Table S3. Experimental environment configuration.

Configuration name	Version and model
System Environment	Python 3.10 (Ubuntu 22.04)
Central Processing Unit	18 vCPU AMD EPYC 9754 128-Core Processor
Image Processor	RTX 3090 (24GB)
Image Acceleration Library	Cuda 12.1
Random Access Memory	60GB
Deep Learning Framework	PyTorch 2.1.0

1.2 Figures

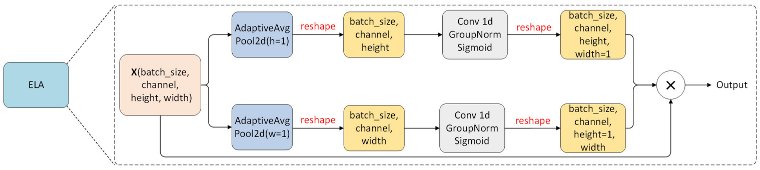

Figure S1. The structure of the ELA (Efficient Local Attention) module.The first reshape operation transforms the feature map from a four-dimensional tensor into a three-dimensional tensor to match the input format of the 1D convolutional layer. The second reshape, performed after the 1D convolution and group normalization processes, is required to restore the processed feature map to the same dimensions as the original input feature map in order to proceed with subsequent element-wise multiplication operations.

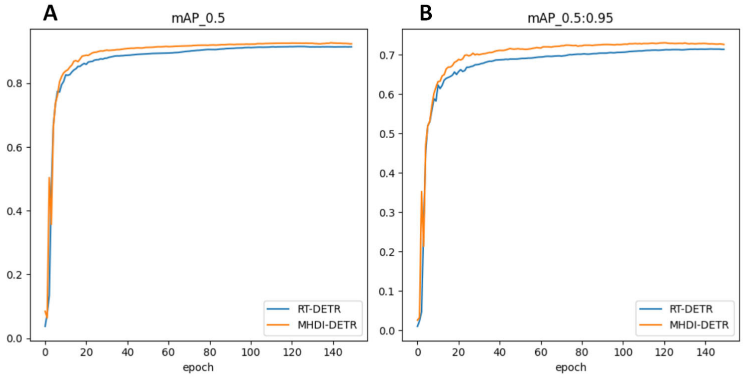

Figure S2. A comparison of average precision curves between the RT-DETR model and the MHDI-DETR model. **(A)** mAP50 training curve. **(B)** mAP50:95 training curve.
